# Major Histocompatibility Complex I Expression by Motor Neurons and Its Implication in Amyotrophic Lateral Sclerosis

**DOI:** 10.3389/fneur.2016.00089

**Published:** 2016-06-13

**Authors:** Giovanni Nardo, Maria Chiara Trolese, Caterina Bendotti

**Affiliations:** ^1^Laboratory of Molecular Neurobiology, Department of Neuroscience, Mario Negri Institute for Pharmacological Research IRCCS, Milan, Italy

**Keywords:** neurodegeneration, motor neuron, SOD1^G93A^ mice, MHC class I, amyotrophic lateral sclerosis

## Abstract

Neuronal expression of major histocompatibility complex I (MHCI)-related molecules in adults and during CNS diseases is involved in the synaptic plasticity and axonal regeneration with mechanisms either dependent or independent of their immune functions. Motor neurons are highly responsive in triggering the expression of MHCI molecules during normal aging or following insults and diseases, and this has implications in the synaptic controls, axonal regeneration, and neuromuscular junction stability of these neurons. We recently reported that MHCI and immunoproteasome are strongly activated in spinal motor neurons and their peripheral motor axon in a mouse model of familial amyotrophic lateral sclerosis (ALS) during the course of the disease. This response was prominent in ALS mice with slower disease progression in which the axonal structure and function was better preserved than in fast-progressing mice. This review summarizes and discusses our observations in the light of knowledge about the possible role of MHCI in motor neurons providing additional insight into the pathophysiology of ALS.

## Introduction

Amyotrophic lateral sclerosis (ALS) is an irreversible, fatal adult onset neurodegenerative disorder causing degeneration of motor neurons in the brain stem, spinal cord, and cerebral cortex. The muscle denervation leads to impairment of the neuromuscular system, resulting in paralysis and death for respiratory failure within 2–3 years after symptom onset. The majority of ALS cases are sporadic, while about 10% are inherited; however, the two forms are clinically and pathologically indistinguishable. A limited number of genes, including SOD1, TARDBP, FUS, C9orf72, and TBK1, are responsible for a significant proportion of familial and sporadic ALS cases (1, 2). So far, mice overexpressing a transgene of human cytosolic superoxide dismutase Cu, Zn dependent (SOD1) with ALS-associated mutations are the animal models that most closely mimics the pathological phenotype of both familial and sporadic ALS (3). The transgenic mouse carrying the hSOD1G93A mutation (SOD1^G93A^), developed more than 20 years ago, has been widely used to unravel the cascade of molecular mechanisms underlying the pathogenesis and progression of ALS (4). These mice develop the disease in adulthood, with progressive muscle wasting and paralysis until death, which is normally induced by euthanasia when they are unable to right themselves within 10 s after being placed on their side. In both mouse models and ALS patients, the denervation of neuromuscular junctions (NMJs) and the axonal retraction occur long before the loss of MN cell bodies in the spinal cord and before the symptoms of neuromuscolar deficit become evident (3, 5–8). It is likely that initially the axons that are still intact can generate compensatory collateral sprouting in order to maintain functional muscle activity, but later, when this compensatory mechanism fails and the motor neuron degeneration progresses, there is an irreversible muscular atrophy (9).

An intrinsic mechanism at the level of motor neuron cell bodies and axon contributes to the pathogenesis of ALS through multiple signaling pathways that have been extensively reviewed (4, 10, 11). However, accumulating evidence indicate that ALS is a non-cell autonomous disease influenced by sustained astrogliosis, with microglial activation and blood-derived immune cells infiltration in the spinal cord that may have critical functions during the disease course (12, 13).

A number of comprehensive overviews have been published on the contribution of the innate and adaptive immune system in ALS pathology; so, this review focuses on the mechanisms of cross-talk between degenerating motor neurons and the adaptive immune cells, particularly on the role of the major histocompatibility complex I (MHCI).

## MHCI in the CNS

An intriguing hypothesis suggests that the evolution of the adaptive immune system may have coincided with elaboration of the neural crest and tissues derived from it, such as the jaws, at the time of increasing complexity of the nervous system (14, 15). In these terms, the adaptive immune system may be an evolutionary offshoot of the vertebrate nervous system (14, 15). This notion is fostered by several genomics studies indicating that the adaptive immune system-related signaling genes were ancestrally involved in the development and/or function of the nervous system (14, 15). In this scenario, it is hardly surprisingly therefore to see how specialized immune cells – T cells, B cells, and dendritic cells – have evolved an immunological synapse structurally and functionally reminiscent of the neural synapse (16, 17).

One of the main factors in the adaptive immune system is the MHCI, classically involved in the presentation of peptides derived from proteolysis mediated by the immunoproteasome (IP) to cytotoxic lymphocytes (18). MHCI belongs to the ever-increasing class of immune proteins (e.g., cadherins; Thy-1; calcineurin; NF-κB, CD22) later detected in the nervous system (19), suggesting that the two compartments share common signal transduction mechanisms. However, some of these molecules, while maintaining the same structure, may also have a different function depending on the context in which they act: a feature termed pleiotropy (20). In the past decade, studies on MHCI activity in the CNS have mainly aimed at demonstrating its role in brain development and plasticity, a topic that has been amply reviewed (21–23). Instead, less information is available about its role in adults. Under normal conditions, the MHCI is weakly or even not expressed in mature neuronal cells, but its expression dramatically increases after viral or parasitic infection (24–26), exposure to cytokines (27–31), and pharmacological manipulation of electrical activity (32). In addition, MHCI becomes activated in neurons targeted to progressive degeneration (33).

Motor neurons seem to be the most responsive in triggering the MHCI during normal aging (34, 35), after insults such as the axotomy (36) or ventral horn root avulsion (37) or during chronic diseases such as experimental autoimmune encephalomyelitis in mice (38). We recently reported that spinal motor neurons of transgenic mice carrying the mutant SOD1^G93A^ markedly activated the expression of several molecules associated with MHCI at the onset and during the progression of the disease (39–41). The mechanism triggering this response and its local consequences are not clearly defined yet. However, these findings open a new scenario in the pathophysiology of ALS as regards cross-talk between the peripheral immune system and CNS, which may be aimed either at preventing or exacerbating the motor neuron loss. It remains to be seen whether the expression of MHCI by MNs is closely related to direct communication with infiltrating immune cells (CD8^+^ T cells) or whether alternative mechanisms can influence motor neuron viability in an immune system-independent manner. In this review, we will comment on both these possibilities trying to define a potential role of MHCI in ALS.

## The Nature and Genetics of MHCI

Major histocompatibility complex class I molecules are trimeric complexes assembled in the endoplasmic reticulum (ER). It consists of two non-covalently linked polypeptide chains, known as α-chain (divided into three domains: α1, α2, and α3) and beta 2 microglobulin (β_2_m) chains that can bind a large set of antigenic peptide fragments (8–11 amino acids) produced in the cytosol by the IP and translocated into the ER through the *transporter associated with antigen presentation* (TAP) (18). The MHCI is then transferred to a vesicle that fuses with the plasma membrane to present the peptide fragment extracellularly to CD8^+^ T lymphocytes. These cells recognize MHCI through the CD8 receptor and the antigenic peptide trough the T cell receptor (TCR).

**Figure 1:**
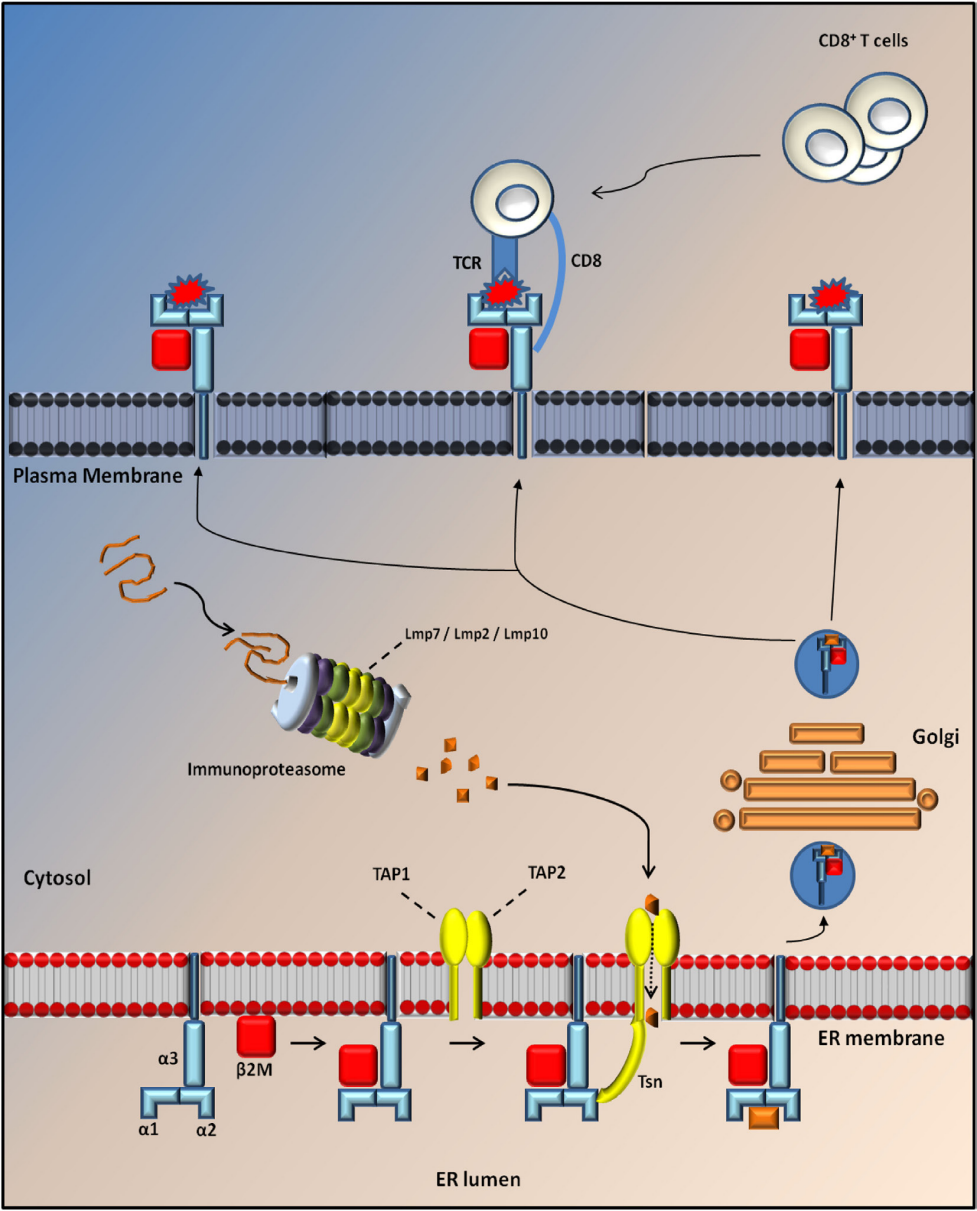
**Antigen processing through MHC I molecules**. MHCI is a tripartite complex that is composed of a non-covalent association of heavy chain (*blue*) with β_2_m (*red*). The third component is represented by a short peptide of 8–10 aa (*orange*), which is processed by the proteasome and translocated to the endoplasmic reticulum (ER) lumen through Tap protein (TAP1; TAP2; *yellow*) where it is loaded onto the MHC complex through the tapasin (Tsn; *yellow*). After loading, MHCI dissociates from TAP/Tsn and traffics to the cell surface along the secretory pathway, where it is monitored by cytotoxic CD8^+^ T cells. Normally, the exposure of a self-peptide on the cell membrane avoids the activation of an immune response. Conversely, the exposure of a non-self-peptide (*red*) induces the activation of an immune response by CD8^+^ T cells with induction of the immunoproteaseome in which three catalytic proteasome β subunits are replaced by three immunosubunits (Lmp7, Lmp2, and Lmp10).

**Figure 2:**
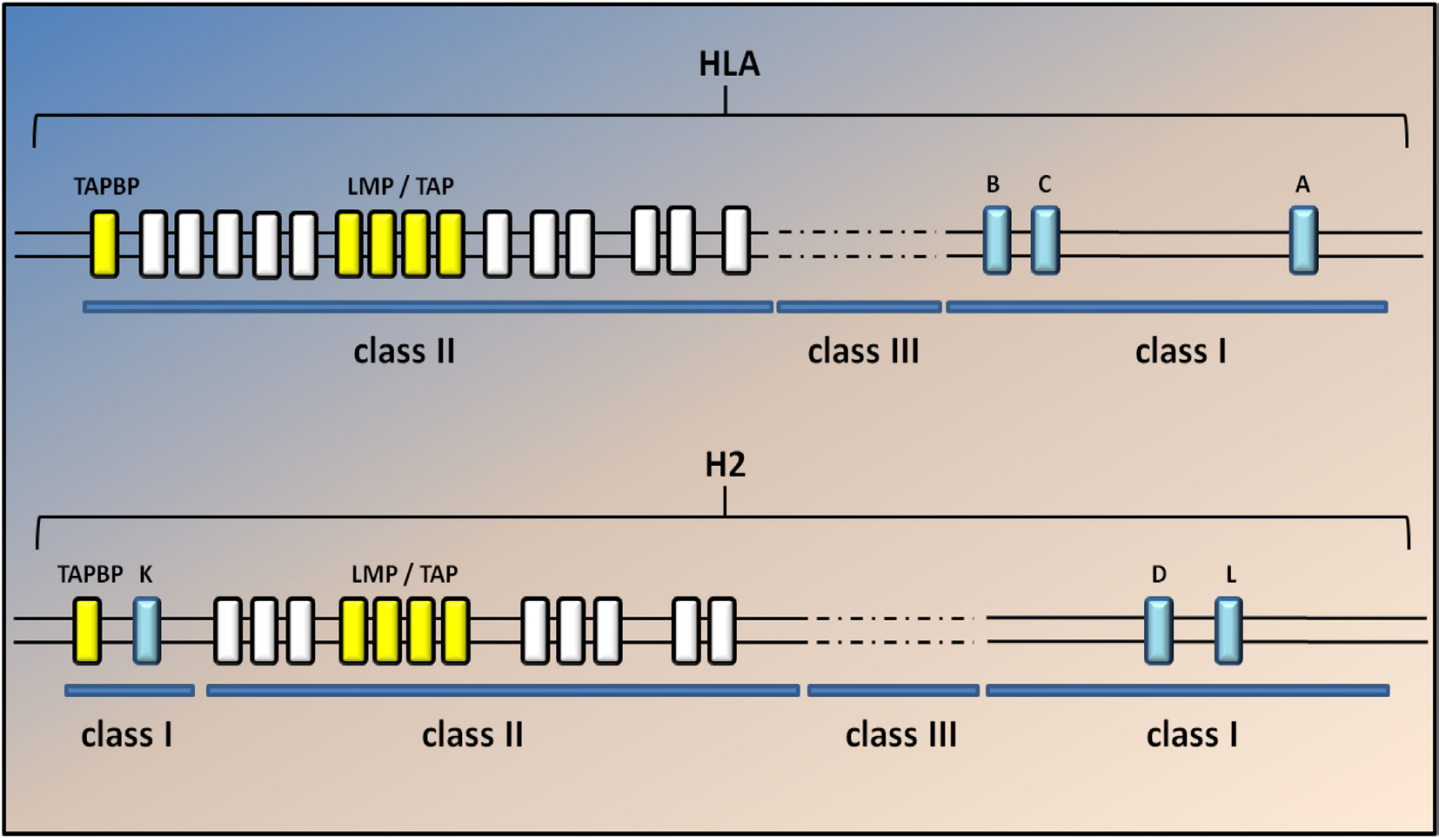
**Genomic map of the human and mouse MHC class I region**. A simplified diagram of the human and mouse MHCI genomic regions shows the MHC spanning approximately 3.6 Mb, located on chromosome 6 in humans and 17 in mice. The classical MHCI genes (blue) are highly polymorphic. Class II and III genes are indicated by white and dashed lines but are not annotated here. The light chain of MHCI molecules, β_2_m, is encoded on a separate chromosome (15 in humans and 2 in mice). Classical MHC class I genes include HLA-A, HLA-B, and HLA-C in humans and H2-K, -D, and -L in mice. H2-L is closely related to H2-D and appears to be present only in the BALB/c mouse strain. The two TAP genes (Tap1 and Tap2; yellow) lie in the MHC class II region close to the immunoproteasome genes (Lmp2 and Lmp7); *yellow* in both humans and mice. The gene for tapasin (Tapbp) lies at the edge of the MHC locus nearest the centromere. The general arrangement of the MHC is similar in humans and rodents, the main difference being that MHCI genes in mice have become separated at both ends of the MHC by class II and III genes.

The composition of the MHCI-associated antigen peptides varies from one cell type to another and can be perturbed by cell-intrinsic and -extrinsic factors including altered cell metabolism and infection (42). Once CD8^+^ T lymphocytes recognize the peptides as non-self or neo-self-antigens, they activate the IP and kill the presenting cells directly through the Fas or perforin pathways and/or indirectly by the release of cytokines (18, 42) (Figure 1). Classical MHCI alpha chains (class Ia) are encoded by three genes in both human (HLA-A, -B, and -C) and mice (H2-K, -D, and -L) located in the MHC locus on chromosomes (chr) 6 and 17, respectively. The gene for β_2_m is located on a different chromosome (chr 15 in humans and chr 2 in mice) (43) (Figure 2).

The MHC region is the most polymorphic of the entire human genome (43). Each HLA locus is present in multiple variants (alleles) that characterize the individual haplotype according to a numerical designation (e.g., HLA-A2, HLA-B5); in mice, each haplotype is identified by a letter (e.g., H-2K^d^, H-2D^k^).

Gene alterations in the MHC class I region have been linked to various disorders with neurological symptoms, including spinocerebellar ataxia, Huntington’s disease, Parkinson’s disease, multiple sclerosis, narcolepsy, dyslexia, schizophrenia, and autism (44–47). This is a product of the role of MHC class I in adaptive immunity, as well as its extraordinary genetic diversity.

A high frequency of the HLA-A3 and HLA-B12 antigens was initially found to be associated with ALS patients with rapid and slow disease progression, respectively. However, this was not validated in a subsequent study comparing the HLA antigenic frequency in ALS patients and control groups (47, 48). Both studies were made on small cohorts of patients and limited to specific geographical areas; so, further analysis is necessary to gain an objective view of the genetic association between the MHCI locus and ALS.

The MHC locus is also extremely polygenic as it includes MHC class Ia genes, MHCII class Ia genes, and non-classical genes for both MCHI and MHCII complexes (class Ib and class IIb) (18, 43). In addition, the two TAP genes (*Tap1* and *Tap2*) lie in the MHC class II region close to the IP genes (*Lmp2* and *Lmp7*), whereas the gene for tapasin (*Tapbp*), a chaperon protein essential for peptide loading, lies at the edge of the MHC locus nearest the centromere (Figure 2). The genetic linkage of the MHC class I genes with *Tap*, *Tapbp*, and *Lmp* genes suggests that the entire MHC region has been selected during evolution for antigen processing and presentation (18). No correlation between genetic variations in the sequence of these genes and ALS has been described yet. Nevertheless, the genetic variant D76N of β_2_m is responsible for a very rare form of familial systemic amyloidosis (49), whereas different genetic variants of the IP component Lmp7 have been associated with many diseases, including viral infection, autoimmune disease, and malignant tumors (50).

## MHCI in the CNS and PNS: Implications for the Immune Response

CNS is considered to be immunoprivileged in order to protect the postmitotic neural cells from the entry of pathogens, circulating immune cells, and other potentially damaging factors that may cause the neuronal injury and death. This is made possible by the presence of a physical blood–brain barrier (BBB) maintained by tight junctions between brain endothelial cells, the basal lamina of these cells, and astrocyte end-feet processes. In addition, cells in the CNS display very little or no expression of immune molecules, such as the MHCI, thus avoiding attack by immunocompetent cells and this contributes to the immunoprivileged status (51). In pathological conditions (viral infection, neuroinflammation, or neurodegeneration), a leakage in the BBB has been reported even before the overt neurodegeneration together with the neuronal upregulation of MHCI, as seen in human postmortem tissues (33, 51, 52). For example, both MHCI and β_2_m are strongly expressed by cortical and hippocampal neurons in Rasmussen’s encephalitis, a childhood disease causing chronic inflammation of the brain with infiltration of T cells in the parenchyma (53). The same occurs in dysmorphic/dysphasic cortical neurons of focal cortical dysplasia, tuberous sclerosis complex, and ganglioglioma cases (54), all diseases caused by infantile tauopathies. In neurodegenerative diseases of adulthood like Parkinson disease, both MHCI and β_2_m were upregulated by dopaminergic neurons in the substantia nigra and in norepinephrinergic neurons of the locus coeruleus in postmortem tissues (33).

**Figure 3:**
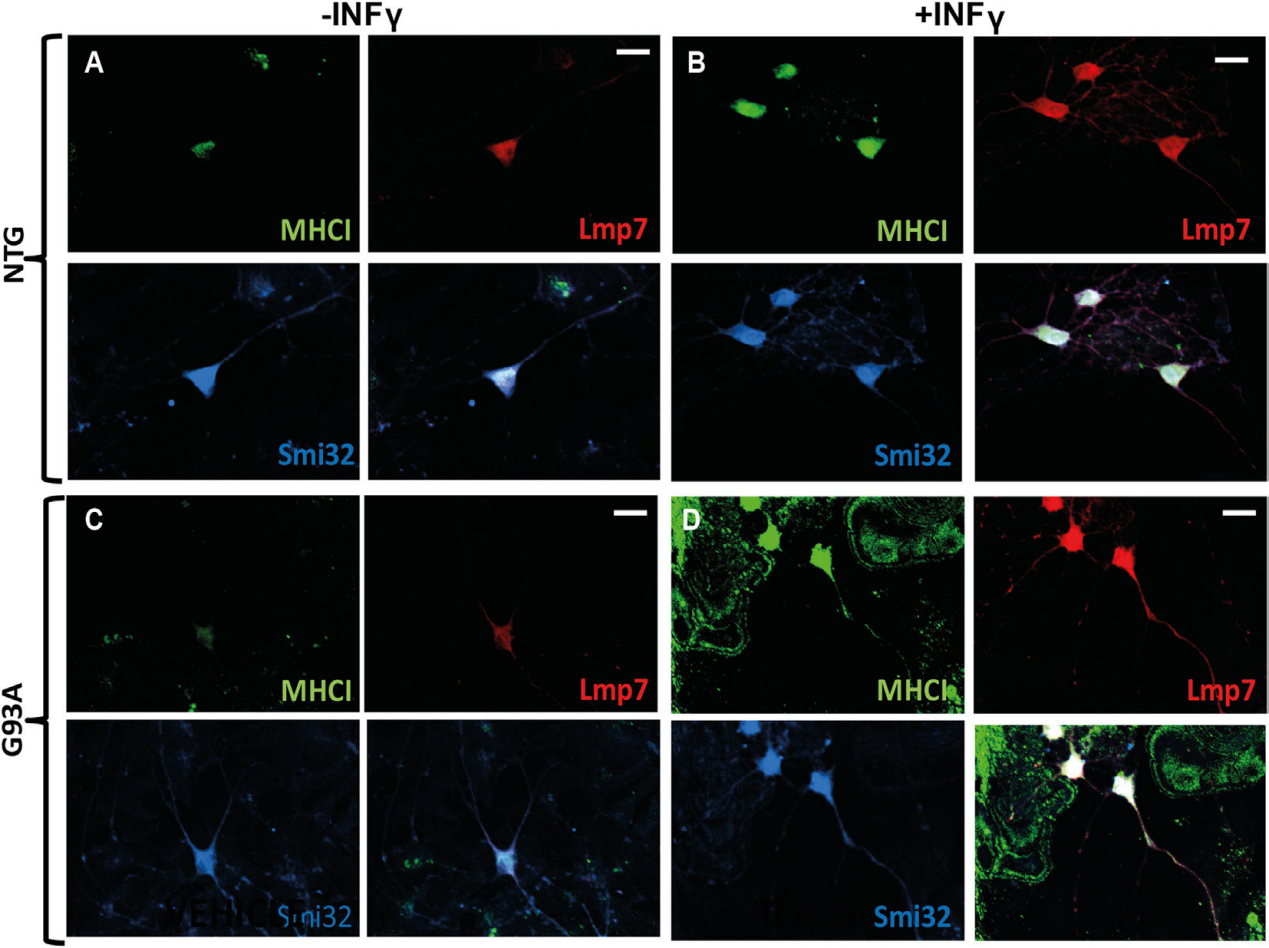
**MHCI and LMP7 are strongly activated in primary motor neurons by INFγ**. **(A–D)** Confocal micrographs, at different magnifications, showing marked activation of MHCI (*green*) and LMP7 (*red*) by primary motor neurons (SMI-32; *blue*) of **(B)** C57 Ntg and **(D)** C57SOD1^G93A^ mice after exposure to INFγ (50 U/ml) for 24 h; scale bar: 50 μm.

More evidence is available on the neuronal expression of MHCI in *in vitro* and *in vivo* models under inflammatory stimuli such as cytokines or viral infection (52). For example, the rat primary motor neurons express high levels of both MHCI heavy chain and β_2_m mRNAs after stimulation for 24, 48, and 72 h, with IFN-γ, which is normally produced by activated T-cells and natural killer (NK) cells *in vivo* (55). Similarly, cultured primary catecholaminergic neurons expressed high levels of MHCI upon exposure for 72 h to IFN-γ, microglial medium prestimulated by LPS or exposure to high levels of l-dihydroxyphenylalanine (l-DOPA) (33). We also observed that INFγ exposure for 24 h, dramatically induced the expression of MHCI and LMP7 in primary spinal motor neurons of both non-transgenic and C57SOD1^G93A^ mice cocultured with non-transgenic primary cortical astrocytes, and the two proteins colocalize in both somata and motor axons (Figures 3A–D).

Strong upregulation of MHCI and related molecules was also seen in the CNS of mice infected with neurotropic viruses. Redwine et al. (26) showed that infection with a strain of murine hepatitis virus induced the transcription of MHCI mRNA by oligodendrocytes, neurons, microglia, and endothelia. Since the virus causes a chronic, inflammatory demyelinating disease, it was suggested that the MHCI activation in neurons was related to classical direct neuron antigen-specific interactions with CD8^+^ T cells leading to damage to the neurons. Studies *in vitro* on primary mouse neurons infected by neurotropic Borna disease virus (BDV) or pulsed by lympho-choriomeningitis virus envelope glycoprotein (GP33) confirmed this indicating that activated CD8^+^ T cells establish stable contacts with axons in an antigen- and MHCI-dependent manner (56–58). These stable Fas (neuronal cells)–FasL (CD8^+^ T cells) contacts rapidly cause segmental damage of neurites in the form of spheroids or beads, independent of detectable morphological changes to the somata (57). Notably, Sauer et al. (59) reported that sustained exposure of primary mouse cortical neurons to IFNγ for 72 h was sufficient to upregulate the axolemmal expression of MHCI H-2Kb molecules competent to present exogenously or endogenously derived SIINFEKL (chicken ovalbumin) peptides. They discovered that cytotoxic T cells isolated from C57BL/6-Tg(TcraTcrb)1100Mjb/J (OT-I) mice and that express a transgenic TCR engineered to recognize H-2Kb-presenting SIINFEKL peptide, formed cytotoxic immune synapses with and injured MHCI-expressing axons, and mediated axon injury through a MHCI and granzyme B-dependent mechanism.

Although the precise mechanism by which CD8^+^ T cells damage neurons calls for further investigation, these data suggest that, if appropriately stimulated, neuronal cells can activate MHCI signaling as a means of communication with cytotoxic T cells.

## MHCI in the CNS and PNS: The Pleiotropic Role

In the past 10 years, the use of animal models has enables us to identify an additional non-immunological function for MHCI in CNS neurons, strictly related to synaptic remodeling and plasticity. More specifically, the lack of a functional MHCI in mice profoundly affects synaptic density and transmission of neuronal cells in hippocampus, visual cortex, and cerebellum during normal development (22). From these studies, it emerges that MHCI is required for the establishment of appropriate connections between neurons. This activity is apparently immune independent and specifically related to the regulation of long-term plasticity of excitatory synaptic transmission. It now appears that MHCI molecules normally inhibit NMDA receptor (NMDAR) function and regulate trafficking of AMPARs after NMDAR stimulation (60). Lee et al. (61) clearly demonstrated that MHCI induces functional and structural synapse pruning during development, balancing the Ca^+^ influx mediated-long terminal potentiation (LTP) in favor of a long terminal depression (LTD) between CNS neurons. The pleiotropic role MHCI was further investigated in adult mice after a mechanical insult or trauma. These studies produced controversial results about the specific role of MHCI in CNS.

Escande-Beillard et al. (62) showed that the addition of picomolar amounts of recombinant MHCI to cultures of wild-type neurons could inhibit neurite outgrowth while transgenic C57Bl/6 mice engineered to express higher levels of self-Db (MHCIa) in their CNS neurons had alterations in hippocampal morphology and retinogeniculate projections, as well as impaired neurorepair responses to CNS insult (63). In keeping with this, the absence of MHCI enhanced neuroprotection after stroke (64). Stroke induced in mice lacking these proteins has smaller infarcts, larger corticospinal axonal projections, fewer reactive astrocytes, and lesser neuronal death supporting the conclusion that MHCI signaling contributes to brain injury after ischemia. Thus, the significant rises in MHCI components after stroke might reduce axonal outgrowth and synaptic plasticity of surviving neurons and circuits, limiting functional recovery.

In contrast, other studies show that MHCI is necessary to preserve the physiological activity of injured motor neurons in the spinal cord, enhancing the recovery of motor function following a mechanical insult. For example, Oliveira et al. (37) reported that the lack of a functional MHCI in *β_2_m*^−/−^ mice subjected to axonal transection strongly influenced synapse density and axonal regeneration of spinal motor neurons. In *β_2_m*^−/−^ mice, the synaptic detachments of the soma of lumbar motor neurons 1 week after peripheral sciatic nerve transection were more extensive than in wild-type animals. This surplus removal selectively involved the clusters of inhibitory synapses without affecting the loss of excitatory synapses, which remained the same in normal and *β_2_m*^−/−^ mice. Thus, after axon lesion motor neurons preferentially remove excitatory inputs, maintaining a subset of inhibitory inputs on the cell and this last effect is strictly dependent on the presence of MHCI. In the same study, Oliveira et al. (37) found that fewer motor neurons were able to regenerate new axons in the distal nerve stump after axotomy in *β_2_m*^−/−^ mice than wild-type mice, suggesting a positive effect of MHCI in axonal regeneration.

This was subsequently confirmed by studies showing that wild-type mouse strains with greater ability to upregulate neuronal MHCI expression after injury had more effective axonal regrowth (65). In addition, transgenic mice with enhanced neuronal MHCI expression (NSE-Db) recovered locomotor function better after spinal cord injury than wild-type mice (66). On the basis of these observations, Thams et al. (36) extensively analyzed MHCI expression and distribution in mice subjected to axotomy. Surprisingly, despite high levels of mRNA expression, MHCI was barely detectable in the motor neurons cell bodies of intact and lesioned motor neurons, although there was a marked increase in immunoreactivity for MHCI in the surrounding microglia. Interestingly, when the peripheral nerve compartment of motor neurons was analyzed, marked MHCI immunoreactivity was found at the level of putative motor axons of transected sciatic nerve and in the NMJs of gastrocnemius muscles.

Thams et al. (36) also reported that mice defective in H2-K and H2-D genes (K^b−/−^; D^b−/−^ mice) had an abnormal pattern of innervation of the hind limb muscles and diaphragm compared with wild-type mice. There was a higher density of acetyl choline receptor (AChR) clusters, disruption of synaptic band structure, and fewer terminal Schwann cells (SCs). These events, which profoundly affect axonal regeneration and muscle reinnervation, are probably responsible for the delayed improvement in grip strength of K^b−/−^ D^b−/−^ versus wild-type mice after sciatic nerve crush (36). These results suggested that, under stress conditions, MHCI molecules as soon as they are synthesized are anterogradely transported in the axon, an event described also for the vomeronasal system *in vivo* (67) and cortical and hippocampal neurons *in vitro* (58, 68). However, it cannot be excluded that MHCI mRNA is transported to the site of injury where it is locally translated (69).

These observations suggest that MHCI plays a role in regulating the density of NMJs during muscle reinnervation. This effect might be due to the inhibition of excessive formation or elimination of inappropriate NMJs during reinnervation. However, the molecular mechanisms of this activity are far from clear, and further studies are necessary to establish the specific role of MHCI in the periphery.

## MHCI in ALS: Implications for the Adaptive Immune Response

In addition to glial cell activation, a hallmark in the CNS of both ALS patients and animal models is the infiltration of blood-derived immune cells (12, 70, 71). This suggests that ALS is characterized by the loss of the immune surveillance typical of the healthy CNS. In fact, altered BBB and spinal cord blood barrier (SCBB) have emerged from the human pathology and various experimental observations in animal models of the disease (72, 73). A relevant role of T cells in the disease process in ALS has been suggested on the basis of marked infiltration of CD8^+^ T cytotoxic and CD4^+^ T helper lymphocytes in the ventral horn of the spinal cord of ALS patients (70, 74). This is corroborated by abnormalities in T lymphocytes and NK cells in blood of ALS patients (75–78), although there are differences among studies that may be explained by the heterogeneity of the ALS cohorts and the limited numbers of patients examined. In ALS animal models too, a progressive and massive increase of the CD8^+^ T and CD4^+^ T cells in the spinal cord has been reported (79, 80). Although CD4^+^ T cells are likely to play a crucial protective role in disease by supporting glia-mediated neuroprotection and slow disease progression in experimental ALS (12, 77, 81), CD8^+^ T lymphocytes may be directly involved in ALS pathogenesis, as recently proposed by Bowerman et al. (82). Cytotoxic T lymphocytes may affect motor neuron survival in different ways. The first involves the Fas ligand protein expressed on the surface of the CD8^+^ T cells interacting with the Fas receptor expressed by the target cells, which triggers apoptosis through the classical caspase cascade (83). Fas receptor is highly expressed by motor neurons expressing the ALS-linked SOD1 mutation *in vivo* and Fas induction by administration of exogenous NO (detanonoate) for 48 h triggers a death pathway in cultured mSOD1 motor neurons (84, 85). On the other hand, upon recognition of the motor neuron by CD8^+^ T cells, cytotoxic granules containing perforin and granzyme may be released into the extracellular space. Perforin is a pore-forming protein that might allow entry into the target cells of granzyme serine proteases that induce caspase activation and cell death (83). Thus, this suggests that CD8^+^ T cells have a direct neurotoxic effect on motor neurons in ALS. How this may happen?

**Figure 4:**
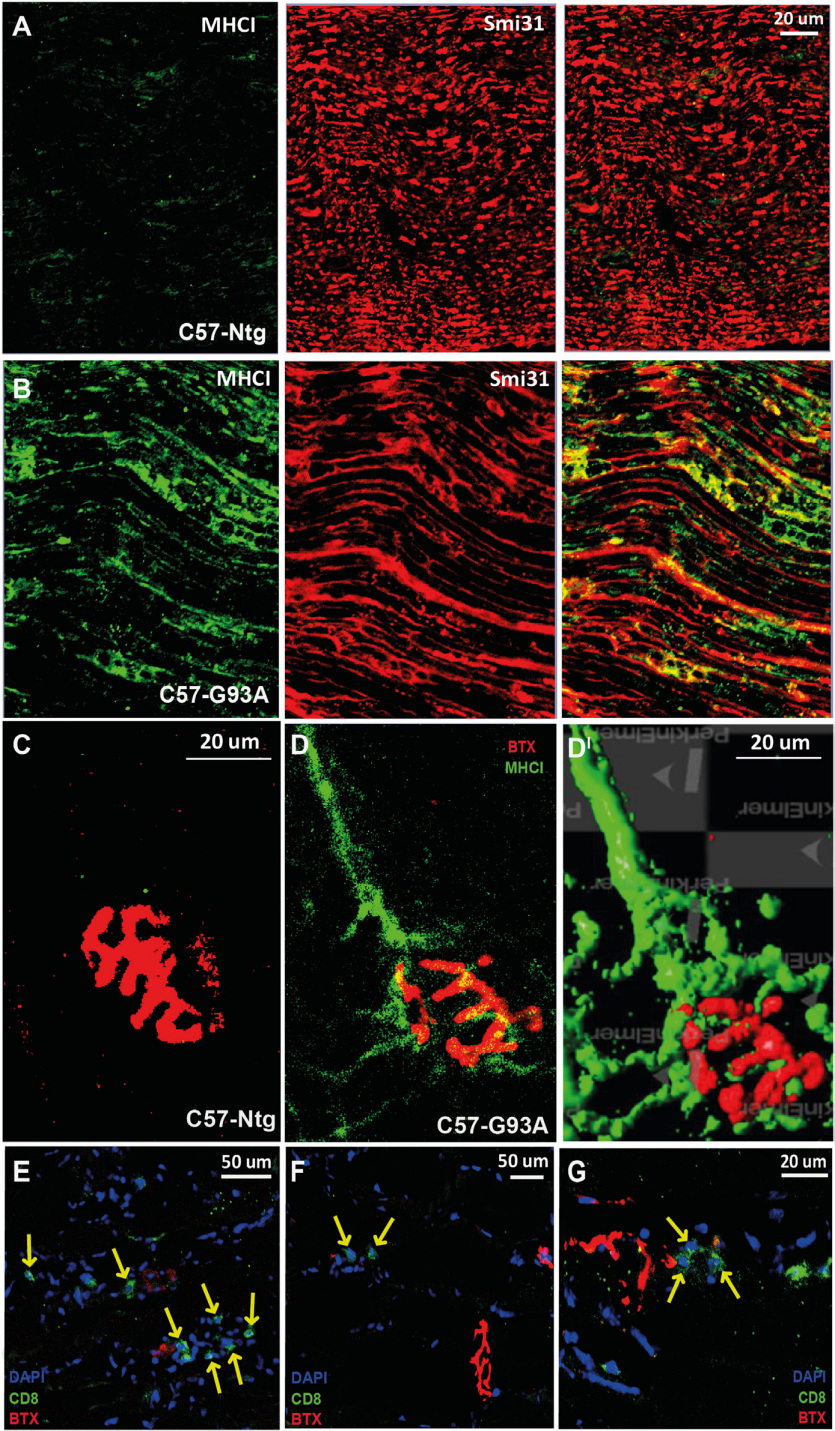
**MHCI activation and CD8^+^ T cell infiltration in the neuromuscular system of SOD1^G93A^ mice at disease onset**. **(A,B)** Confocal micrographs of the sciatic nerve of C57SOD1^G93A^ mice at disease onset showing remarked upregulation of MHCI (*green*) and colocalization with Smi31 (axonal marker; *red*) in pathological conditions. **(C,D^I^)** Confocal micrographs from *tibialis anterior* muscle of **(C)** Ntg and **(D)** C57-G93A mice showing accumulation of MHCI at the NMJ under pathological conditions (BTX; α-bungarotoxin; postsynaptic marker). **(D^I^)** 3D rendering of **(B)** from the same angulation (Volocity – Perkin Elmer). **(E–G)** Confocal micrographs showing the presence cytototoxic T cells (CD8, *green*) in the tibialis anterior muscle of C57-G93A mice in proximity to NMJs, indicated by *yellow arrows*.

We recently reported that the motor neurons of C57SOD1^G93A^ mice at the disease onset markedly upregulate the expression of MHCI, which accumulate preferentially in motor axons and NMJs [Ref. (41) and Figures 4A–D^I^]. In concomitance with this response, there was an upregulation of the IP subunits in the C57SOD1^G93A^ motor neurons, particularly *Lmp7*, suggesting that motor neurons locally produce and expose to the environment an MHCI complex “loaded” with autoantigenic peptides (41). The intense immunolabeling of MHCI protein in the sciatic nerve of SOD1^G93A^ mice indicates a potential direct interaction between motor axons exposing autoantigenic peptide and infiltrating cytotoxic T cells at this level.

The peripheral interaction of motor neurons with cytotoxic CD8^+^ T cells may therefore be the direct cause of motor axon injury in SOD1^G93A^ mice; however, how the MHCI is triggered in ALS motor neuron is not known. A possible explanation lies in the proteinopathy and neuroinflammation typical of this disease (39). The accumulation of misfolded proteins in the motor neurons due to genetic mutations or their oxidation is not only one of the main neuropathological hallmarks of ALS but probably also the *primum movens* in the cascade leading to motor neuron degeneration. The misfolded proteins, which cannot be properly degraded by the constitutive proteasome or other protein quality control systems, can be directed toward the INFγ activated IP (39). At the same time, the misfolded proteins in the motor neurons may trigger the inflammation through the release of danger-associated molecular pattern molecules (DAMPs), including ROS, HMGB1, and HSPs, which activate glial and immune cells to produce inflammatory cytokines including IFNγ (86, 87). IFNγ may then induce upregulation of the IP in the motor neurons, and other cells leading to the overproduction of neuronal and glial antigens presented on MHCI molecules. In primary cocoltures of astrocytes and spinal neurons from transgenic SOD1^G93A^ or non-transgenic mice, the addition of INFγ to the medium for 24 h markedly induce the expression of MHCI and LMP7 in motor neurons and the two proteins colocalize in both somata and motor axons (Figures 3A–D). No differences were observed in the basal expression of MHCI and LMP7 between SOD1^G93A^ and non-transgenic MNs. This indicates that the only presence of mutant SOD1, even if misfolded, is not sufficient to trigger the activation of MHCI cascade in the motor neurons. Since INFγ is secreted by activated microglia and CD8^+^ T cells, the presence of these cells *in vivo* may be a prerequisite to activate the MHCI cascade in SOD1^G93A^ motor neurons triggering the cascade leading to their death. Although the activation of resident microglia may result from the release of DAMPs in the extracellular space around motor neurons, the recruitment of CD8^+^ T is probably a consequence of a disruption of the BBB, which has been reported in ALS patients and mouse models of the disease (72, 73). This scenario suggests the involvement of autoimmunity as a potential pathogenic mechanism of ALS (88). In fact, large-scale mass spectrometry studies have revealed that the repertoire of endogenous peptides that is normally presented by MHCI to CD8^+^ T cells (*self*-MHCI peptides) is more complex and plastic than previously anticipated. Its composition varies from one cell type to another and can be perturbed by cell-intrinsic and -extrinsic factors including dysregulation of cellular metabolism and infection (42). Accordingly, the pro-inflammatory environment of spinal cord and the dysregulated protein metabolism of motor neurons during ALS may promote the activation of the IP and the exposure, on the cell membrane, of antigenic peptides recognized as *non-self* by CD8^+^ T cells, which then activate a cytotoxic autoimmune response.

That said, the contribution of cytotoxic T cells to the pathophysiology of ALS certainly calls for deeper investigations.

However, recent observations do not support the detrimental role of MHCI activation in ALS motor neurons, but rather suggest a protective effect. For example, the MHCI cascade is only barely activated in the motor neurons of 129SvSOD1^G93A^ mice, which have more aggressive disease course and shorter lifespan than C57SOD1^G93A^ mice, although they overexpress the same amount of the mutant SOD1 transgene and show similar loss of lumbar spinal cord motor neurons perikarya (41, 89). The difference in disease severities is probably related to a functional impairment of the motor neurons at the axonal level, as demonstrated by the marked swelling and vacuolation of myelinated axons observed in the ventral spinal cord of the fast-progressing 129SvSOD1^G93A^ mice at the presymptomatic stage, while this was seen later in the slow progressors C57SOD1^G93A^ mice (89). In addition, earlier abnormalities were seen in the axonal compartment of 129SvSOD1^G93A^ mice by *ex vivo* diffusion tensor imaging and histological analysis of the white matter of the spinal cord (90). Axial diffusion was reduced in the ventro-lateral and -dorsal white matter of 129SvSOD1^G93A^ mice compared with the non-transgenic littermates already at the onset of the disease, while in C57SOD1^G93A^ mice, this appeared only at the advanced stage (90).

These data suggest that the activation of MHCI in motor neurons of C57SOD1^G93A^ mice at the onset of the disease may be predictive of better preservation of axonal function, with a positive impact on the disease progression (41). This hypothesis was recently strengthened by Staats et al. (91) who reported that the ubiquitous removal of β_2_m in SOD1^G93A^ mice shortened the disease duration by anticipating their death.

As previously shown, MHCI is mainly expressed by motor axons in the periphery of C57SOD1^G93A^ mice (41), so there may be a privileged interaction between motor neurons and CD8^+^ T cells at this level and not in the spinal cord. The activity of the immune system in the PNS of mSOD1 mice has hardly been considered in ALS, but there is mounting evidence that the contribution of the inflammatory response in the PNS stands in stark contrast with that of the CNS, where the activity of nearby cells (microglia, astrocytes, and infiltrating immune cells) exacerbate cell death and damage by releasing toxic pro-inflammatory mediators over a long period of time. Successful axonal regeneration depends on the coordinated effort of non-neuronal cells that, while removing motor axon debris, release extracellular matrix molecules, cytokines, and growth factors that support axon regrowth (92). New evidence indicates that innate and adaptive immune responses can facilitate CNS repair at a later stage by restricting a prominent secondary wave of damage in multiple sclerosis and other disorders (93, 94). For example, autoimmune T cells that are specific for the myelin basic protein (MBP) protect CNS neurons from secondary degeneration possibly through antigen-mediated production of nerve growth factors into the environment (95). *In vivo* imaging in a mouse ALS model indicated distinct inflammatory activity of CNS microglia compared with PNS macrophages, the latter lacking any substantial morphological reaction during degeneration of peripheral motor axons, but a passive role in clearing debris, particularly lipid-rich myelin (96). This is in line with Chiu et al. (79) who reported an increase in CCL2 expression and intense deposits of complement C3 throughout the parenchyma of the sciatic nerve of SOD1^G93A^ mice which resulted in progressive macrophage infiltration without the activation of classical pro-inflammatory factors such as TNFα and IL-6. CCL2-mediated neuron–macrophage interaction is critical for amplification and maintenance of enhanced regenerative capacity by preconditioning peripheral nerve injury (97). In addition, Barrette et al. (98) found that peripheral macrophages were essential to promote the production of neurotrophins within the regenerating PNS since macrophage depletion significantly slowed axon regeneration and functional recovery after sciatic nerve injury. At the same time, the CCL2-mediated neuron–macrophage interaction is critical for amplification and maintenance of enhanced regenerative capacity by preconditioning peripheral nerve injury (97).

Finally, in keeping with the presence of MHCI at the NMJ of C57SOD1^G93A^ mice, we found T cell infiltrated in close proximity to motor end plates in the *tibialis anterior* muscles of C57SOD1^G93A^ at the disease onset (Figures 4E–G). Therefore, we cannot exclude an interaction between infiltrating T cells and autoantigens, exposed by MHCI on degenerating NMJs, actively influencing synaptic refinement and plasticity. At this same level, CD8^+^ T cells may act in concert with activated peripheral macrophages recruited within bundles of innervating axon tracts of *tibialis anterio*r muscles of SOD1^G93A^ mice at disease onset (79). This suggests that the activity of immune cells may be essential in the degradation of defective terminals in order to facilitate more efficient collateral reinnervation by surviving motor neurons during the first phases of the disease. This is consistent with denervation and reinnervation changes observed in the fALS animal model and ALS patients during the progression of the disease (7).

## MHCI in ALS: The Non-Immunological Role

As discussed previously, MHCI has been firmly implicated in neuronal plasticity, regulating synaptic density and axonal regeneration in the CNS and PNS during development and in brain diseases (22, 33). The mechanisms governing these effects are unknown, but evidence from different compartments of the cerebral cortex indicates the presence of immune-like MHCI receptors in the CNS (22, 23, 52). In fact, although there is no evidence of TCRα and TCRβ expression, CD3ζ is found throughout the CNS and CD3ϵ is expressed in the cerebellum (22). At the same time, NK receptors, such as PirB and Ly49, have been observed in axonal growth cone and synapses of hippocampal neurons *in vivo* and cortical neuron *in vitro* (22). Animal models defective in PirB, CD3ζ, or CD3ϵ show neurological defects similar to those in MHCI-KO mice models, so an interaction of these receptors with MHCI is possible, although not directly proven (22, 52). In the brain, MHCI protein is expressed by neurons, especially on the surface of axons and dendrites (32) as well as both pre- and postsynaptically (99, 100). However, it is almost undetectable in spinal motor neurons soma of axotomized and SOD1^G93A^ mice, but marked accumulation has been seen in motor axons and NMJs (36, 41). Given this MHCI protein expression in the periphery, SCs expressing PirB on their surface may be their counterpart at this level (36, 101). This finding provides a basis for possible MHCI-mediated signaling between motor neuron and SCs in the sciatic nerve involved in motor axon homeostasis and stability.

In the PNS, SCs are the main source of paracrine support to motor axons forming close bidirectional relationship with their neuronal partners. During development, the SCs are essential for the survival of motor neurons, while the neuron-derived factors are essential for the survival and differentiation of SCs along axons (102). These interactions are also important when there is peripheral damage to preserve the myelin sheath around motor axons. SCs, with macrophages, phagocize their myelin sheaths and then SCs enter a de-differentiation/proliferation program to support the motor axon by remyelinating it (103, 104). Early morphological, biochemical, and molecular alterations have been observed in SCs from sciatic nerve of SOD1^G93A^ mice (5, 105–107), suggesting that these cells are dysregulated during the development of ALS. Therefore, in the sciatic nerve, MHCI may potentiate the proliferative activity of SCs during disease progression to promote long-lasting preservation of motor axons. At the same time, the high expression of MHCI at the NMJs may be responsible for the activity of terminal SCs, since the removal of MHCI has a dramatic effect on their proliferation (36). Terminal SCs provide stability, produce growth factors, and are involved in synapse homeostasis (108–110). In addition, they are essential for the removal of inappropriate presynaptic terminals during reinnervation (111, 112).

Major histocompatibility complex I negatively regulates the initial establishment of connections and promotes the elimination of inappropriate synapses in hippocampal and cortical neurons (22). This process is mediated by MHCI also in the NMJs (35). Both developmental synapse elimination and aging-related synapse loss are promoted by MHCI. The reduction of MHCI in the NMJs induces the formation multiply innervated muscle fibers, suggesting that MHCI is essential for the postsynaptic site transition from dual- to monoinnervation. All muscles, during development, undergo a transient period of poly-innervation, receiving as many as 12 distinct inputs from different MNs in the spinal cord at birth (110, 113). These initially superabundant inputs are removed in a protracted process called synapse elimination until each muscle fiber receives input from a single MN.

The muscle reinnervation after a nerve lesion or during the progression of ALS undergoes the same processes of poly-innervation, pruning, and refinement as in normal development (69, 114). Signals promoting axonal growth and branching are activated during this phase. At the same time, growth control mechanisms are turned on to consolidate connection patterns and confine plastic phenomena to specific sites (69). MHCI signaling probably belongs to this second class of mechanisms acting to fine regulate and accelerate collateral reinnervation and sprouting, as a compensatory response to the progressive motor neuron injury ALS. Work on this hypothesis may provide clues on how to develop strategies for increasing the muscle reinnervation and delay the disease progression.

## Conclusion

Over the past 10 years, it has become clear that neuronal MHCI molecules play a significant role in the development and plasticity of the CNS and PNS in normal and pathological conditions (22, 33). Here, we report a protective role of MHCI in ALS, as we observed specific activation of the MHCI pathway in spinal motor neurons of slowly progressing C57SOD^G93A^ mice with MHCI protein accumulating preferentially in motor axons and NMJs. MHCI expression was almost absent in 129SvSOD1^G93A^ mice that have a more aggressive disease due to earlier alterations of motor axons (41).

Our results support previous findings in axotomized mice, indicating that MHCI is fundamental in order to enhance locomotor function and accelerate axonal regrowth and muscle reinnervation in the PNS. In addition, it was recently observed that the ubiquitous removal of β_2_m in SOD1^G93A^ mice shortened the disease duration (91).

Despite the clear importance of MHCI in axonal regrowth and in delaying ALS progression, the mechanisms of these effects are mostly unknown. Here, we speculate about a potential dual activity of neuronal MHCI in the PNS of SOD1^G93A^ mice.

**Figure 5:**
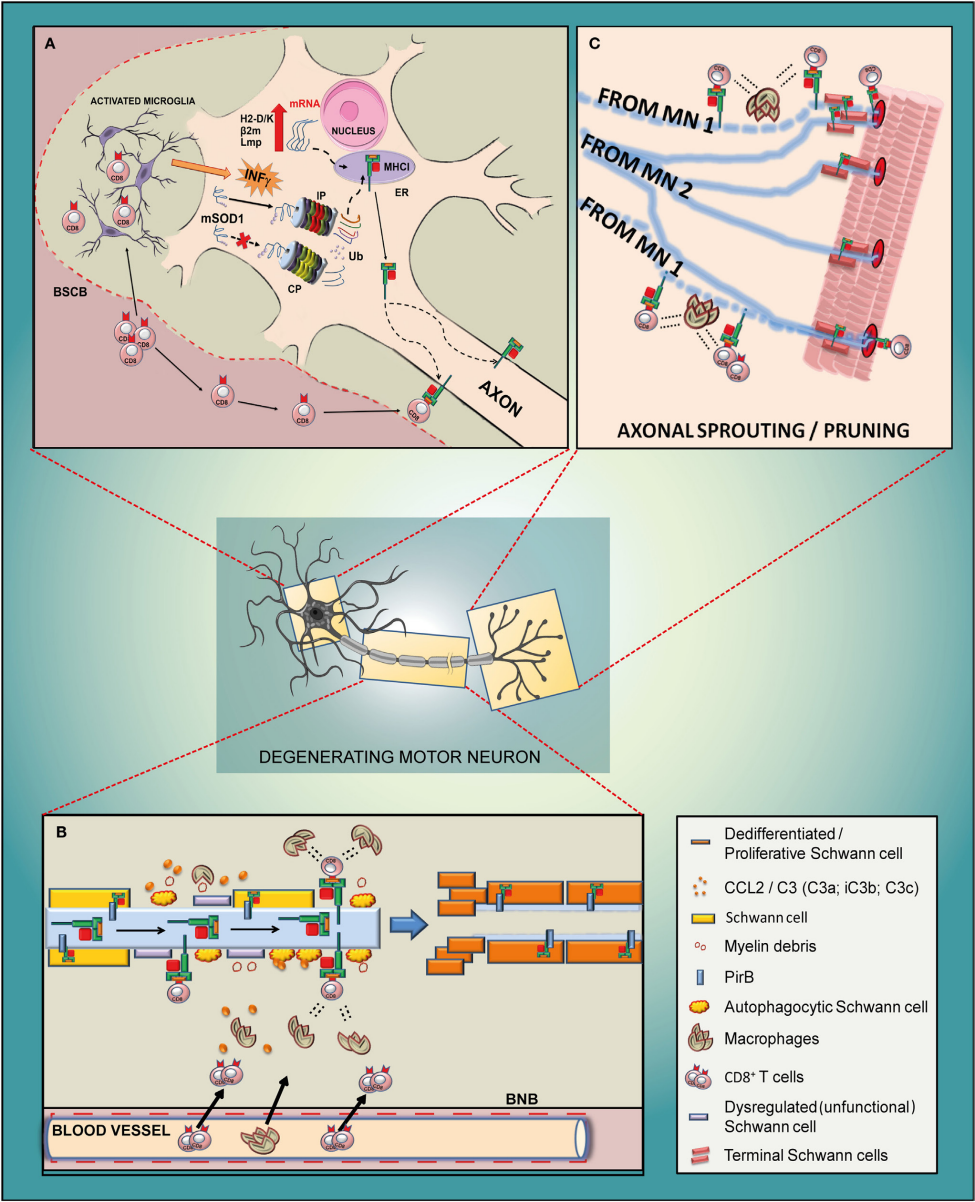
**Schematic diagram of the potential mechanism of action of MHCI-associated molecules in the motor neuron of a mutant SOD1 mouse model of ALS**. **(A)** *Cell body*. Due to an impairment of constitutive proteasome (CP), the mSOD1 (or other misfolded proteins) is driven to the immunoproteasome (IP), which is activated by the INFγ derived from activated resident microglia or from blood-derived CD8^+^ T cells recruited through the impaired blood spinal cord barrier (BSCB). The small antigenic peptides resulting from the IP activity are then loaded onto MHCI and rapidly translocated to the axons. It is likely that the translocation happens after the assembly of the MHCI molecule and peptide, although part of it may also occur along the motor axons. **(B)** *Sciatic nerve*. The antigenic peptide is presented through MHCI by defective motor axons to cytotoxic CD8^+^ T lymphocytes that infiltrate the sciatic nerve together with peripheral macrophages due to the damaged blood nerve barrier (BNB). The concerted removal of defective myelin by dysregulated/autophagocitic SCs, cytotoxic T cells, and macrophages is a prerequisite for the de-differentiation/proliferation of SCs (probably promoted by a motor axons–MHCI/SCs–PirB interaction) in order to create a growth-permissive milieu for new neurites. **(C)** *Neuromuscular junction*. The high expression of MHCI in the motor axon terminals might promote the pruning and collateral reinnervation of muscles through two mechanisms such as (i) by presenting antigenic peptides to cytotoxic T cells which, in concert with macrophages, prune the damaged axon terminals (motor neuron 1; MN 1) and promote collateral reinnervation (MN 2); (ii) by promoting the proliferation of terminal SCs that produce growth factors, provide stability to terminal synapse homeostasis and participate in the removal of inappropriate presynaptic terminals during reinnervation.

The first involves a direct interaction of motor axons with infiltrating CD8^+^ T cells through possible specific self-antigens presented by MHCI in motor neurons. Our *in vitro* and *in vivo* data testify of concomitant activation of the IP and MHCI in mSOD1 motor neurons. In SOD1^G93A^ mice with an accumulation of MHCI in the peripheral nerves and NMJs, this interaction with CD8^+^ T cells may have a central role in regulating the homeostasis of the PNS during disease progression. The second involves the activity of MHCI to directly control the proliferation of SCs in the sciatic nerve and NMJ, in order to preserve the maximal efficiency of motor axon connectivity with target muscles. These immune and pleiotropic activities of MHCI may be closely connected. It is likely that during the course of ALS, the MHCI cascade is activated by motor neurons and transported to the periphery where the recruitment of cytotoxic T cells and peripheral macrophages potentiates the degradation of defective motor fibers (115) expressing axon growth inhibitors (e.g., myelin debris) in an attempt to promote the de-differentiation and proliferation of SCs and the resulting preservation of motor axons connections with muscles (Figures 5A–C).

In the future, it will be crucial to determine what prevailing role MHCI has in motor neurons affected by ALS, whether protective or harmful. Particularly, evaluating which antigens are presented by the damaged MHCI-expressing motor axons to CD8^+^ T cells may allow to finely modulate the cytotoxic activity of these cells during the course of the disease. At the same time, understanding the molecular basis of MHCI at NMJ or in the cross-talk with non-immune cells, such as SCs, may pave the way toward strategies to promote axonal regeneration and muscle reinnervation that may prevent the disease progression.

## Ethics Statement

All procedures involving animals and their care were carried out in accordance with the ethical standards procedure of the Mario Negri Institute, which are in accordance with national (D.L. no. 116, G.U. suppl. 40, February 18, 1992, no. 8, G.U., 14 July 1994) and international laws and policies (EEC Council Directive 86/609, OJ L 358, December 12,1987; National Institutes of Health Guide for the Care and Use of Laboratory Animals, US National Research Council, 1996).

## Author Contributions

GN wrote the manuscript and edited figures. MT wrote part of the manuscript. CB wrote the manuscript and supervised the final version.

## Conflict of Interest Statement

The authors declare that the research was conducted with no commercial or financial conflict of interest.
